# Omega-3 Polyunsaturated Fatty Acids Mitigate Palmitate-Induced Impairments in Skeletal Muscle Cell Viability and Differentiation

**DOI:** 10.3389/fphys.2020.00563

**Published:** 2020-06-03

**Authors:** Bill Tachtsis, Jamie Whitfield, John A. Hawley, Nolan J. Hoffman

**Affiliations:** Exercise and Nutrition Research Program, Mary MacKillop Institute for Health Research, Australian Catholic University, Melbourne, VIC, Australia

**Keywords:** n-3 PUFA, EPA, DPA, DHA, saturated fatty acid, myoblast, myotube, mitochondria

## Abstract

Accumulation of excess saturated free fatty acids such as palmitate (PAL) in skeletal muscle leads to reductions in mitochondrial integrity, cell viability and differentiation. Omega-3 polyunsaturated fatty acids (n-3 PUFAs) such as eicosapentaenoic acid (EPA) and docosahexaenoic acid (DHA) counteract PAL-induced lipid accumulation. EPA and DHA, as well as the n-3 PUFA docosapentaenoic acid (DPA), may therefore mitigate PAL-induced lipotoxicity to promote skeletal muscle cell survival and differentiation. C2C12 myoblasts were treated with 50 μM EPA, DPA, or DHA in the absence or presence of 500 μM PAL for 16 h either prior to myoblast analysis or induction of differentiation. Myoblast viability and markers of apoptosis, endoplasmic reticulum (ER) stress and myotube differentiation capacity were investigated using fluorescence microscopy and immunoblotting. High-resolution respirometry was used to assess mitochondrial function and membrane integrity. PAL induced cell death via apoptosis and increased protein content of ER stress markers BiP and CHOP. EPA, DPA, and DHA co-treatment maintained cell viability, prevented PAL-induced apoptosis and attenuated PAL-induced increases in BiP, whereas only DPA prevented increases in CHOP. PAL subsequently reduced protein content of the differentiation marker myogenin and inhibited myotube formation, and all n-3 PUFAs promoted myotube formation in the presence of PAL. Furthermore, DPA prevented PAL-induced release of cytochrome c and maintained mitochondrial integrity. These findings demonstrate the n-3 PUFAs EPA, DPA and DHA elicit similar protective effects against PAL-induced impairments in muscle cell viability and differentiation. Mechanistically, the protective effects of DPA against PAL lipotoxicity are attributable in part to its ability to maintain mitochondrial respiratory capacity via mitigating PAL-induced loss of mitochondrial membrane integrity.

## Introduction

Excess cellular accumulation of lipids such as palmitate (PAL) is well appreciated to negatively impact skeletal muscle cell viability. PAL is a dietary saturated fatty acid abundant in Western diets that is commonly used to experimentally assess the lipotoxic effects of elevated saturated fatty acids in tissues such as skeletal muscle (Alsabeeh et al., 2018). It has been demonstrated that PAL exposure *in vitro* negatively impacts skeletal muscle cell metabolism by impairing insulin sensitivity (Hage Hassan et al., 2012), suppressing protein synthesis (Perry et al., 2018), and upregulating proteolytic machinery (Woodworth-Hobbs et al., 2014). Furthermore, PAL has been shown to induce endoplasmic reticulum (ER) stress, which consequently activates the unfolded protein response (UPR), a series of coordinated signaling networks that collectively stimulate adaptive responses to re-establish cellular homeostasis (Deldicque et al., 2010). In the event the UPR is unable to restore protein homeostasis in response to a cellular insult such as PAL, programmed cell death can be induced via apoptosis (Bohnert et al., 2018).

In contrast to saturated fatty acids such as PAL, omega-3 polyunsaturated fatty acids (n-3 PUFAs) possess anti-inflammatory properties and can improve skeletal muscle function and metabolism by altering cellular membrane lipid composition (Di Girolamo et al., 2014; Herbst et al., 2014; Mcglory et al., 2016; Jeromson et al., 2017; Gerling et al., 2019). Furthermore, the n-3 PUFA docosahexaenoic acid (DHA) has been shown to ameliorate lipotoxic effects of PAL in skeletal muscle cell models by restoring insulin sensitivity (Bryner et al., 2012) and preventing activation of the UPR in differentiated skeletal muscle myotubes (Woodworth-Hobbs et al., 2014). In contrast to DHA, eicosapentaenoic acid (EPA) is the only n-3 PUFA shown to protect against the deleterious effects of inflammation (Magee et al., 2008) and PAL exposure (Saini et al., 2017) by partially restoring the regenerative capacity of skeletal muscle. EPA and DHA are commonly found in fish oil supplements and have been demonstrated to improve markers of myogenic differentiation (i.e., myosin 4 expression and myotube fusion index) (Briolay et al., 2013).

Docosapentaenoic acid (DPA) is a less-studied n-3 PUFA, however, it possesses similar bioactive properties to EPA and DHA (Kaur et al., 2010, 2016). Cell based studies have shown that DPA is an intermediate n-3 PUFA and can be readily converted to EPA (Achard et al., 1995; Kaur et al., 2011a; Norris and Dennis, 2012), while conversion to DHA is limited (Kaur et al., 2010). However, it remains to be determined whether DPA can also protect skeletal muscle against cellular insults such as PAL in a similar manner to these other n-3 PUFAs. Moreover, previous studies examining the effects of n-3 PUFAs in skeletal muscle cell models *in vitro* have utilized fully differentiated myotubes (Kamolrat and Gray, 2013; Tachtsis et al., 2018), while little emphasis has been placed on comparing their effects in proliferating myoblasts and during the induction of myotube differentiation.

The primary aim of this study was to therefore compare the efficacy of the n-3 PUFAs EPA, DPA, and DHA in mitigating PAL-induced lipotoxicity in skeletal muscle cells. The secondary aims were to determine if n-3 PUFAs attenuate PAL-induced lipotoxic cellular mechanisms including ER stress induction and loss of mitochondrial integrity, as well as subsequent defects in myotube differentiation. We hypothesized that EPA, DPA, and DHA would elicit similar protective effects against PAL-induced lipotoxicity and attenuate PAL-induced impairments in myotube formation to a similar extent.

## Materials and Methods

### C2C12 Cell Culture

Mouse C2C12 myoblasts (Lonza, Basel, Switzerland) were cultured in DMEM containing 4.5 g/L glucose and L-glutamine (Life Technologies, Carlsbad, CA, United States) supplemented with 10% fetal bovine serum (Life Technologies, Carlsbad, CA, United States) and 0.5% penicillin/streptomycin (Life Technologies, Carlsbad, CA, United States) in T75 cm^2^ flasks at 37°C in a humidified 5% CO_2_ incubator. At 70% confluency, myoblasts (passage numbers 7–14) were washed with 1 × PBS and trypsinized using 0.25% trypsin solution (Life Technologies, Carlsbad, CA, United States). Dissociated cells were washed with 1 × PBS solution (Life Technologies, Carlsbad, CA, United States), centrifuged (Heraeus Sepatech Megafuge 1.0R centrifuge, BS4402/A rotor #3360) at 3000 g for 3 min and re-seeded at a density of 3.0 × 10^5^ mL onto 6 or 96 well plates for immunoblotting and fluorescence microscopy analysis. Each biological replicate experiment was performed with cells cultured and harvested independently, and where applicable technical replicates were also included within each biological replicate (as described in each analysis below).

### Preparation of Fatty Acid Treatments

DHA (cis-4, 7, 13, 16, 19-docosahexaenoic acid), EPA (cis-5, 8, 11, 14, 17-eicosapentaenoic acid) and PAL were obtained from Sigma-Aldrich (St. Louis, MO, United States). DPA (cis-7, 10, 13, 16, 19-Docosapentaenoic acid) was purchased from Nu-Chek Prep Inc (Elysian, MN, United States). Stock solutions of n-3 PUFAs and PAL were prepared by dissolving each fatty acid in 100% (v/v) ethanol. Working solutions of n-3 PUFAs and PAL were made by diluting each stock solution in pre-warmed DMEM containing 2% (w/v) fatty acid free BSA (Sigma-Aldrich, St. Louis, MO, United States) to achieve a final concentration of 50 μM for each n-3 PUFA (Woodworth-Hobbs et al., 2017; Jeromson et al., 2018) and 500 μM for PAL (Perry et al., 2018). PAL stock solutions were sonicated at 50°C for 10 min to ensure PAL was completely solubilized. For cell treatments, working solutions were prepared fresh on the day of each experiment. All fatty acids were incubated at 37°C for approximately 30 min before being applied to cells. A final concentration of ethanol (<0.01%) was used as a vehicle control and was identical between each treatment. Control treatment groups also contained 2% (w/v) fatty acid free BSA. The number of cells initially plated for each experiment and treatment condition was equal. At an equal level of 70% confluency between each treatment condition, cells were incubated with either vehicle, EPA, DHA, DPA and/or co-treated with PAL for 16 h. For assessment of differentiation capacity following this single overnight 16 h n-3 PUFA and/or PAL treatment(s), the following morning media was switched to DMEM containing 2% horse serum (Life Technologies, Carlsbad, CA, United States) to induce myotube differentiation. During the 5-day period of myotube differentiation, media was replaced every 2 days.

### n-3 PUFA Incorporation

After 16 h treatment with 50 μM EPA, DPA or DHA, media was aspirated and myoblasts were washed twice with ice-cold 1 × PBS, pH 7.4. 100 μL of trypsin EDTA was added to each well and incubated at 37°C for 5 min. Cells from 3 wells of a 6-well plate were pooled together and 1.5 mL PBS was added to trypsinized cells. The cells were then transferred to 10 mL glass screw-capped (Teflon-lined) tubes, and lipids were extracted using a 2:1 chloroform:methanol mixture, as previously described in Kaur et al. (2011b) and Portolesi et al. (2007). In brief, after the addition of chloroform:methanol mixture, the samples were vortexed for 10 min and centrifuged (Heraeus Sepatech Megafuge 1.0R centrifuge, BS4402/A rotor #3360) at 1500 g for 10 min to separate the aqueous and organic phases at room temperature. The organic phase containing the lipid was removed and transferred to a new glass tube and evaporated under a stream of nitrogen. Fatty acids were derivatized with 2% H_2_SO_4_ in 100% methanol for 3 h at 80°C to form the fatty acid methyl esters (FAME). The purified FAMEs were separated by capillary gas liquid chromatography (GLC) using a 50 m 0.32 mm (I.D.) fused silica bonded phase column (BPX70, SGE, Melbourne, Australia). Fatty acids were identified by comparison with standard mixtures of FAME as detailed by Kaur et al. (2011b), and results were calculated using response factors derived from FAME standards of known composition (Nu-Chek Prep, Inc., Elysian, MN, United States) and normalized to cell number.

### Cell Viability Analysis

Following n-3 PUFA and/or PAL treatment(s), myoblasts were trypsinized (described above) and equal volumes of cell suspension and 0.4% trypan blue (Life Technologies, Carlsbad, CA, United States) were combined. To assess the number of remaining viable cells in each condition, 10 μL of this combined solution was loaded into an automated cell counting system (Countess II FL; Life Technologies, Carlsbad, CA, United States). DAPI staining (1:20,000, Life Technologies, Carlsbad, CA, United States) was also used to assess cell viability following n-3 PUFA and/or PAL treatment(s), as morphological changes to nuclei following PAL treatment are typically observed during the late stages of cell death (Mandelkow et al., 2017). Nuclei were defined as DAPI positive areas ranging from 10 to 1000 μm^2^. By defining these size criteria, small nuclear fragments along with large nuclei clusters were manually excluded. The nuclear morphology parameters analyzed include area (μm^2^), perimeter (μm), and brightness (relative fluorescence units/nuclei). A total of 10 fields were randomly selected from each treatment condition, and images were collected at 20× magnification. For all treatment conditions, parameters such as brightness and contrast were kept consistent during the acquisition and analysis of all images. Analysis was performed by converting images to 16-bit grayscale images and applying the threshold function within ImageJ software.

### TUNEL Assay

To assess apoptosis following n-3 PUFA and/or PAL treatment(s), TUNEL assay was performed using an In Situ Direct DNA Fragmentation (TUNEL) Assay Kit (Abcam, Cambridge, United Kingdom, ab66108) according to the manufacturer’s instructions. Briefly, cell smears after paraformaldehyde fixation, blocking and permeabilization were incubated with TUNEL reaction mixture for 1 h at 37°C protected from light and counterstained with RNase/PI solution for 30 min. Cells were imaged using the EVOS FL Auto 2 Cell Imaging System at 20× magnification. A total of 10 fields were randomly selected for imaging, and for each treatment condition TUNEL positive cells relative to total cells in these fields were counted (Life Technologies, Carlsbad, CA, United States). For all treatment conditions, parameters such as brightness and contrast were kept consistent during the acquisition and analysis of all images. Analysis was performed by converting images to 16-bit grayscale images and applying the threshold function within ImageJ software.

### Immunofluorescence Analysis of Myotube Differentiation

To assess myotube formation at 5 days following 16 h overnight n-3 PUFA and/or PAL treatment(s) and induction of differentiation the following morning, cells were washed twice with ice-cold 1× PBS, fixed with 100% ethanol and permeabilized with 0.1% Triton X-100. Cells were blocked overnight with 2% BSA solution and incubated with desmin primary antibody (D33, MA5-13259, Life Technologies, Carlsbad, CA, United States) overnight at 4°C. Cells were then incubated for 2 h at room temperature in Alexa Fluor 488 secondary antibody (1:200, Life Technologies, Life Technologies, Carlsbad, CA, United States) protected from light. Nuclei were counterstained with DAPI. Images were captured using EVOS FL Auto 2 Cell Imaging System (Life Technologies, Carlsbad, CA, United States) at 10× magnification. A total of 5 fields were randomly selected from each treatment condition. For all treatment conditions, parameters such as brightness and contrast were kept consistent during the acquisition and analysis of all images. Analysis was performed using the manual cell counter function within ImageJ.

### Immunoblotting

Following n-3 PUFA and/or PAL treatment(s) myoblasts and myotubes were rinsed twice with ice-cold 1× PBS and scraped in the presence of lysis buffer containing 50 mM Tris HCl, 1 mM EDTA, 1 mM EGTA, 10% glycerol, 1% Triton X-100, 50 mM NaF, 5 mM sodium pyrophosphate, 1 mM DTT, 10 μg⋅mL^–1^ trypsin inhibitor, 2 μg⋅mL^–1^ aprotinin, 1 mM benzamidine, and 1 mM PMSF (Sigma-Aldrich, St. Louis, MO, United States). Lysates were passed through a 22-gauge needle ten times for homogenization and centrifuged (Eppendorf 5424 R centrifuge, F45-24-11 rotor) at 15,871 g for 15 min at 4°C to remove cellular debris. The resulting supernatant was analyzed for total protein concentration using a BCA protein assay (Pierce, Rockford, IL, United States). Lysates were re-suspended in Laemmli sample buffer at 0.33 μg⋅μL^–1^. Proteins were separated by SDS-PAGE using 4–20% Stain-Free Precast gels (Bio-Rad, Hercules, CA, United States) and transferred to polyvinylidene fluoride membranes, blocked with 5% non-fat milk, washed with 10 mM Tris⋅HCl, 100 mM NaCl, and 0.02% Tween 20 (Sigma-Aldrich, St. Louis, MO, United States) and incubated overnight at 4°C with primary antibody as specified by the respective supplier (1:1000, unless stated otherwise) including BiP (Cell Signaling Technology, Danvers, MA, United States, #3177, clone number C50B12), CHOP (Cell Signaling Technology, Danvers, MA, United States #5554, clone number D46F1), phospho-eIF2α Ser51 (Cell Signaling Technology, Danvers, MA, United States #9721), total eIF2α (Cell Signaling Technology, Danvers, MA, United States #9722), myosin heavy chain (Thermo Fisher Scientific, Waltham, MA, United States, PA5-31466), and myogenin [F5D] (Abcam, Cambridge, United Kingdom, ab1835). Membranes were then incubated with secondary antibody (anti-rabbit, HRP-linked, Cell Signaling Technology, Danvers, MA, United States 1:2000). All gels were equally loaded with 10 μg protein in each well. An identical pooled sample of cell lysate was run on every gel for each target analyzed to normalize band intensity between gels, and all proteins were normalized to the total protein loaded in each lane using stain-free imaging technology (Bio-Rad, Hercules, CA, United States) as performed previously (Tachtsis et al., 2016; Smiles et al., 2019).

### Mitochondrial Respiration

C2C12 myoblasts were incubated with 2% BSA (control), DPA or co-treated with PAL and DPA for 16 h. High-resolution respirometry experiments were performed in MiR05 respiration medium (0.5 mM EGTA, 3 mM MgCl2, 60 mM K-lactobionic acid, 20 mM taurine, 10 mM KH_2_PO_4_, 20 mM HEPES, 100 mM D-Sucrose and 1g/L BSA, pH 7.1) at 37°C using an Oxygraph-2k FluoRespirometer (Series G) and DatLab Version 7.3 (Oroboros Instruments, Innsbruck, Austria). For all experiments, each chamber was loaded with 500 μL respiration medium containing 1 × 10^6^ viable cells (determined using a hemocytometer), which were permeabilized by the addition of 4.1 μM digitonin. Respiration was initiated with 10 mM pyruvate and 5 mM malate, followed by 5 mM ADP. Glutamate (10 mM) and succinate (10 mM) were added to determine maximal respiration. Mitochondrial membrane integrity was tested through the addition of cytochrome c (10 μM), with <15% increase in respiration indicating preservation of outer mitochondrial membrane integrity (Johnson et al., 2015; Leckey et al., 2018). Finally, maximal electron transfer was stimulated through the titration of CCCP (sequential 0.5 μM additions). All experiments were performed in duplicate from cells of the same passage (technical replicates), with respiration rates averaged from both chambers of one Oxygraph-2K instrument. All treatments (with and without palmitate) and conditions (control and DPA) were performed on the same day. Cells were grown and harvested independently to generate a total sample size of 4 biological replicates. Each biological replicate experiment consisted of 2 technical replicate measurements using separate chambers. For all experiments, rates of oxygen consumption (*J*O_2_) were normalized to chamber volume (2 mL).

### Statistical Analysis

Statistical analysis was conducted using Sigma Plot (Version 12.5). Data were analyzed by a one-way or two-way analysis of variance (ANOVA). Where significant main effects were detected, Bonferroni *post hoc* analysis (condition × treatment) were used to determine differences within each group. All data are presented as mean ± SEM with *p*-values < 0.05 considered statistically significant. All results are representative of a minimum of 3 biological replicate experiments from independently cultured and harvested cells.

## Results

### EPA, DPA, and DHA Protect Against PAL-Induced Cell Death

**FIGURE 1:**
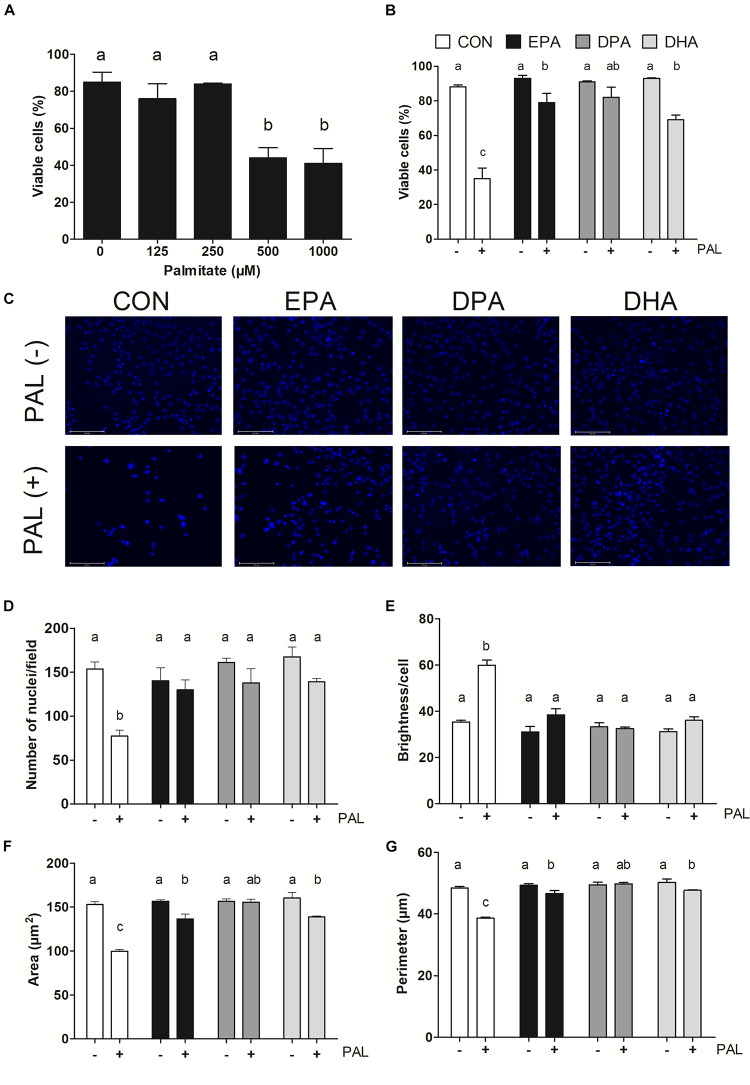
EPA, DPA, and DHA protect against PAL-induced reductions in cell viability. Trypan blue exclusion test dose response experiment in myoblasts following 16 h of palmitate (PAL) treatment to assess cell viability **(A)**. Trypan blue exclusion test of cell viability following treatment with 500 μM PAL and/or 50 μM EPA, DPA, or DHA **(B)**. Representative images of nuclear DAPI staining following treatments **(C)**. Average number of nuclei/field **(D)**, brightness/nuclei **(E)**, nuclear area **(F)**, and nuclear perimeter (μm) **(G)**. Treatments that do not share letters are considered statistically significant (*p* < 0.05; myoblasts were cultured and harvested independently to generate a total sample size of 3–4 biological replicates). Scale bar represents 125 μm. Values are presented as mean ± SEM.

Myoblasts were treated for 16 h with PAL (0–1000 μM dose response) to identify the concentration(s) at which PAL-induced lipotoxicity occurs. The lowest concentration tested at which significant reductions in cell viability occurred was 500 μM (∼48% reduction compared to CON *p* < 0.05), and therefore this concentration was used in all subsequent experiments (Figure 1A). There was no difference in viability observed between individual n-3 PUFAs alone relative to untreated control (CON) cells, while treatment of CON cells with PAL (CON-PAL) induced a ∼60% reduction in viability (*p* < 0.05) (Figures 1B,C). When myoblasts were co-treated with EPA or DHA in the presence of PAL, cell viability was significantly increased relative to CON-PAL (∼55 and 50%, respectively, *p* < 0.05), although cell viability remained ∼10 and 20% lower than CON cells, respectively (*p* < 0.05). In contrast, co-treatment with DPA prevented PAL-induced decreases in cell viability, as DPA-PAL cell viability was not significantly different from CON or DPA cells alone.

Nuclear staining with DAPI confirmed treatment with PAL induced cell death, as evidenced by reductions in the number of nuclei per field (∼50%, *p* < 0.05; Figure 1D), nuclear area (∼35%, *p* < 0.05; Figure 1F) and perimeter (∼20%, *p* < 0.05; Figure 1G) versus CON. An increase in nuclear brightness (∼70% relative to CON, *p* < 0.05) was detected following PAL exposure, as demonstrated to be indicative of cell death (Mandelkow et al., 2017; Figure 1E). However, there were no detectable changes between any of the PUFA treatments alone or PAL-PUFA co-treatments relative to CON in terms of nuclei number or brightness (Figures 1D,E). Both EPA and DHA attenuated PAL-induced decreases in nuclei area (*p* < 0.05; Figure 1F) and perimeter (*p* < 0.05; Figure 1G) relative to CON-PAL, although these indices were decreased relative to CON and PUFA alone. In contrast, DPA co-treatment completely protected against PAL-induced reductions in cell area and perimeter, as there were no differences detected in these measures between DPA-PAL compared to CON or DPA alone (Figures 1F,G).

**FIGURE 2:**
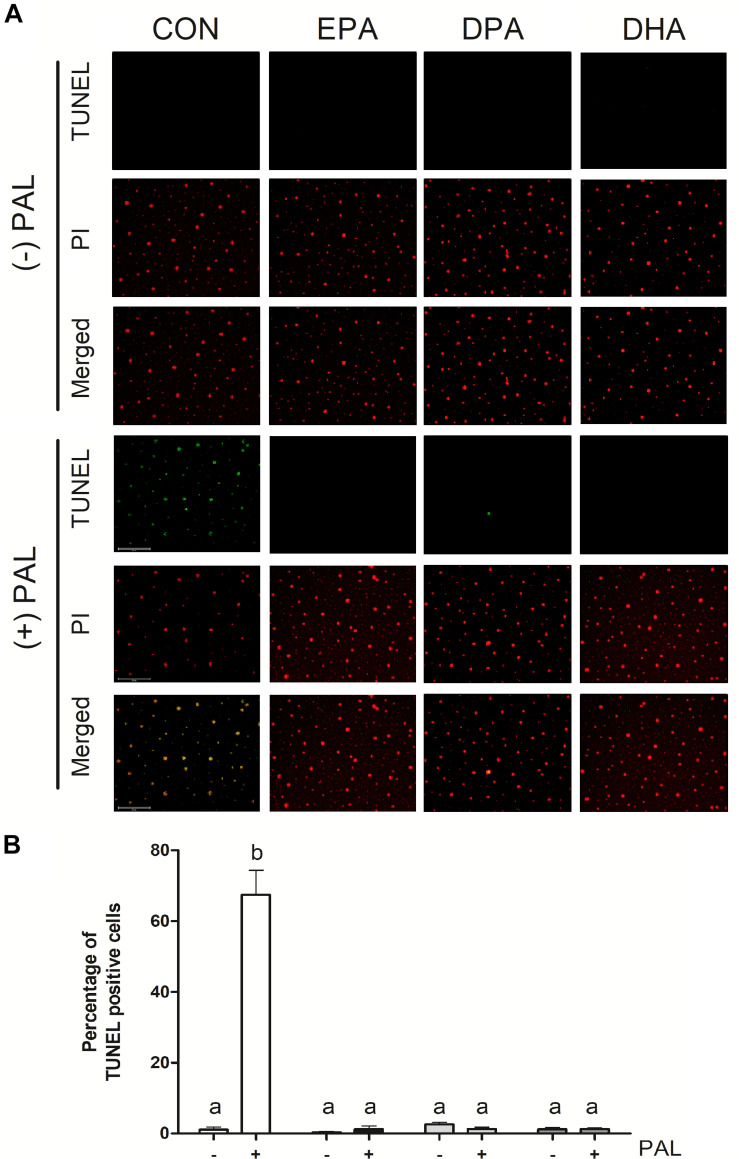
EPA, DPA, and DHA attenuate PAL-induced increases in DNA fragmentation characteristic of apoptosis. Representative images of myoblast TUNEL staining 16 h following treatment with 500 μM PAL and/or 50 μM EPA, DPA or DHA **(A)**. Percentage of TUNEL positive cells relative to total number of cells in the field **(B)**. Treatments that do not share letters are considered statistically significant (*p* < 0.05; myoblasts were cultured and harvested independently to generate a total sample size of 3 biological replicates). Scale bar represents 125 μm. Values are presented as mean ± SEM.

TUNEL assay staining of DNA fragmentation characteristic of the late phases of apoptosis confirmed that PAL treatment increased apoptosis [∼60-fold (∼6000%) increase in the % of TUNEL-positive cells relative to CON, *p* < 0.05]. PAL-induced apoptotic cell death was reduced in the presence of EPA, DPA and DHA by ∼60-fold (∼6000%), ∼51-fold (∼5100%), and ∼54-fold (5400%) relative to CON-PAL, respectively, with no detectable differences observed between PUFA treatments alone or PAL-PUFA co-treatments relative to CON (Figures 2A,B).

### EPA, DPA, and DHA Intracellular Concentrations Are Increased in Myoblasts After 16 h Treatment

The intracellular concentration of EPA increased by ∼17-fold (∼1700% relative to CON, *p* < 0.05) following EPA treatment (Supplementary Figure S1A). DPA treatment increased intracellular DPA concentration by ∼4.2-fold (∼427% relative to CON, *p* < 0.05). DPA treatment also increased intracellular EPA concentration by 1.8-fold (∼180%) relative to CON that was not significantly different from DPA concentration (*p* = 0.062), indicating retro-conversion of DPA to EPA (Supplementary Figure S1B). DHA treatment increased DHA intracellular concentration by ∼2.5-fold (256% relative to CON, *p* < 0.05; Supplementary Figure S1C).

### EPA, DPA, and DHA Attenuate PAL-Induced Increases in the ER Stress Marker BiP, but Only DPA Prevents PAL-Induced Increases in CHOP

**FIGURE 3:**
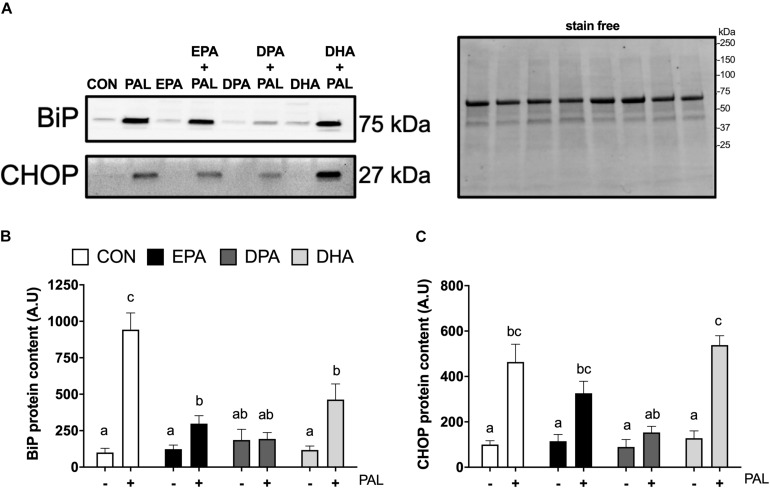
EPA, DPA, and DHA attenuate PAL-induced increases in the ER stress markers BiP and CHOP. Representative images of ER stress proteins BiP and CHOP following 16 h treatment with 500 μM PAL and/or 50 μM EPA, DPA or DHA **(A)** and representative image of a stain free blot used for normalization shown with molecular weight markers indicated. Quantified protein content of BiP **(B)** and CHOP **(C)**. In the absence of PAL, little to no CHOP protein was detectable. All densitometry values are expressed relative to protein content determined by stain free imaging and presented in arbitrary units relative to CON. Treatments that do not share letters are considered statistically significant (*p* < 0.05; myoblasts were cultured and harvested independently to generate a total sample size of 5 biological replicates). Values are presented as mean ± SEM.

As ER stress has been established to be involved in PAL-induced reductions in cell viability and potential mitigation by n-3 PUFAs (Perry et al., 2018; Chen et al., 2020), markers of ER stress signaling were next analyzed in myoblasts to determine whether EPA, DHA, and DPA also potentially attenuated ER stress induction responses to PAL. Relative to CON, increases in BiP [∼8.4-fold (∼840%), *p* < 0.05; Figures 3A,B] and CHOP [∼3.6-fold (∼360%), *p* < 0.05; Figures 3A,C] protein content were observed with PAL treatment. Co-treatment with EPA, DPA or DHA attenuated PAL-induced increases in BiP by ∼70, 80, and 50%, respectively, compared to PAL (*p* < 0.05). However, only DPA prevented the PAL-induced increase in BiP protein content (Figure 3B).

PAL-induced increases in CHOP protein content were still observed with EPA and DHA co-treatments [∼64% and ∼3.2-fold (∼320%), respectively (*p* < 0.05), compared to the respective n-3 PUFA treatment alone]. In contrast, CHOP protein content in DPA treated myoblasts remained at similar levels to CON following PAL co-treatment (Figure 3C). There was also a tendency for DPA to prevent the PAL-induced increase in CHOP expression (∼66% reduction compared to PAL, *p* = 0.06; Figure 3C). No changes in the ER stress marker eIF2α Ser51 phosphorylation or total protein content were detected between any of the PAL and/or n-3 PUFA treatments (Supplementary Figures S2A–D).

### EPA, DPA, and DHA Ameliorate the Deleterious Effects of PAL on Myoblast Differentiation

**FIGURE 4:**
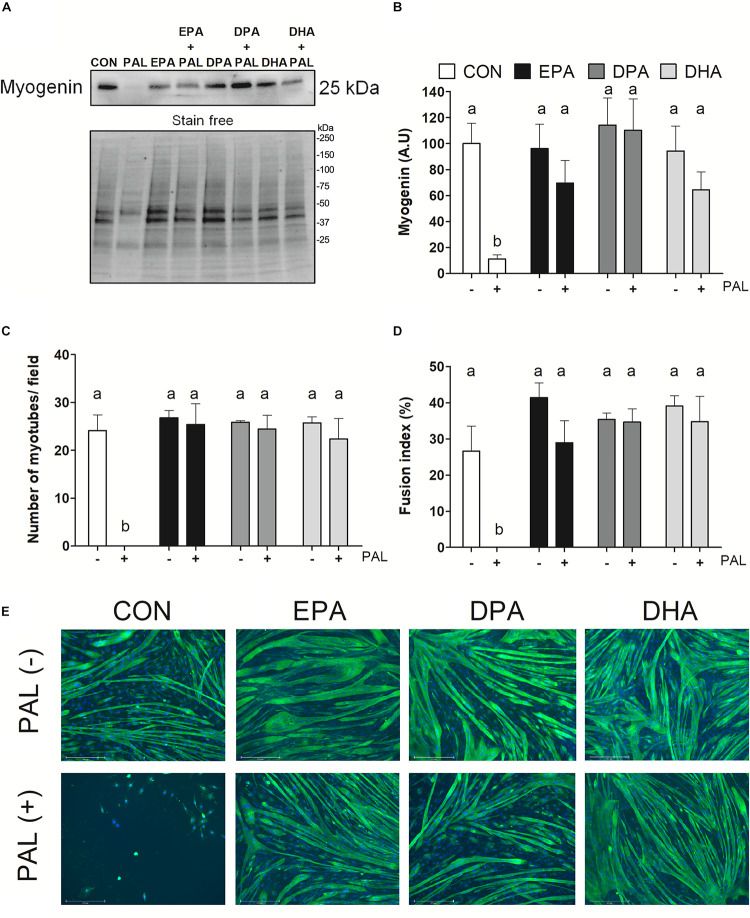
EPA, DPA, and DHA protect against the inhibitory effects of PAL on myotube differentiation. Protein expression of the myogenic protein myogenin 120 h following treatment with 500 μM PAL and/or 50 μM EPA, DPA, or DHA, with a representative image of a stain free blot used for normalization shown with molecular weight markers indicated **(A)**. Quantified protein expression of the myogenic protein myogenin **(B)**. All densitometry values are expressed relative to protein content determined by stain free imaging and presented in arbitrary units relative to CON. Treatments that do not share letters are considered statistically significant (*p* < 0.05; *n* = 6 biological replicates). Average number of myotubes/field **(C)** and fusion index (number of nuclei in tubes relative to the total number of nuclei expressed as a percentage) **(D)**. Representative images of each treatment group at 120 h (*n* = 3 biological replicates) **(E)**. Scale bar represents 275 μm. Values are presented as mean ± SEM.

To next investigate the effects of n-3 PUFAs on subsequent induction of muscle cell differentiation, myoblasts were differentiated for 5 days following exposure to the single treatment of PAL and/or each n-3 PUFA (EPA, DPA, or DHA) and myogenic markers were assessed. Protein content of the myogenic marker myogenin was reduced following PAL treatment (∼90% reduction compared to CON, *p* < 0.05; Figures 4A,B). In contrast, EPA, DPA, and DHA each protected against the deleterious effects of PAL on differentiation, as indicated by increased myogenin protein content (∼50, 110, and 46%, respectively, *p* < 0.05 compared to CON-PAL; Figure 4B). Consistent with these findings and the observed reductions in myoblast viability, PAL prevented the subsequent formation of myotubes (Figures 4C–E). However, myoblasts treated with EPA, DPA, and DHA effectively differentiated to myotubes following PAL co-treatment, with no changes observed in the average number of myotubes per field or myotube fusion index between each n-3 PUFA treatment either in the absence or presence of PAL (Figures 4C–E).

### DPA Protects Against PAL-Induced Impairments in Mitochondrial Membrane Integrity Without Altering Mitochondrial Content or Maximal Mitochondrial Respiration

**FIGURE 5:**
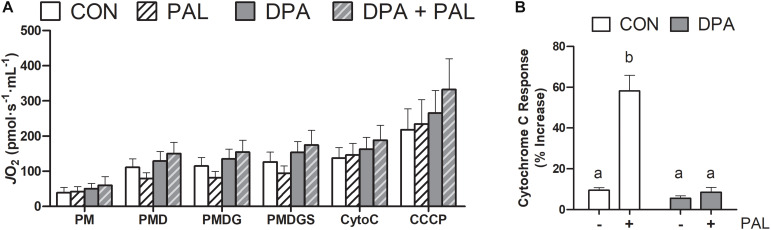
Treatment with DPA and/or PAL does not alter mitochondrial respiration, but DPA maintains mitochondrial membrane integrity in response to PAL. In **(A)**, *J*O_2_ represents mitochondrial O_2_ flux. Cytochrome c retention test response expressed as (PMDGS-CytoC/CytoC) × 100 **(B)**. Treatments that do not share letters are considered statistically significant (*p* < 0.05). Myoblasts were cultured and harvested independently to generate a total sample size of 4 biological replicates. Values are presented as mean ± SEM.

**FIGURE 6:**
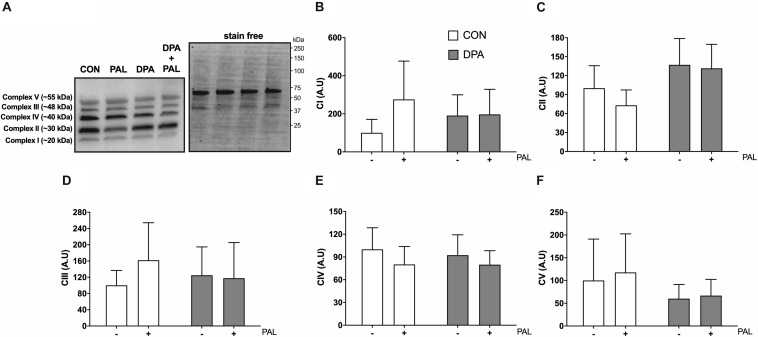
Treatment with DPA and/or PAL does not alter myoblast mitochondrial content. Protein content of mitochondrial OXPHOS complexes following 16 h treatment with 500 μM PAL and/or 50 μM EPA, DPA, or DHA and representative images **(A–F)**. All densitometry values are expressed relative to protein content determined by stain free imaging (**A**; representative image shown with molecular weight markers indicated) and presented in arbitrary units relative to CON. Myoblasts were cultured and harvested independently to generate a total sample size of 6 biological replicates. Values are presented as mean ± SEM.

Given the known role of mitochondrial cytochrome c in the induction of cell death via apoptosis, mitochondrial function and content were evaluated following treatment with PAL and/or DPA, which provided the most robust attenuation of PAL-induced cell death and ER stress markers. Non-ADP-stimulated (pyruvate + malate; PM), ADP-stimulated (PMD), maximal complex I supported respiration (PMDG), maximal coupled (PMDGS), and uncoupled (CCCP) respiration were unchanged with PAL and/or n-3 PUFA treatment (Figure 5A). However, CON myoblasts treated with PAL exhibited a significant increase in mitochondrial respiration when exogenous cytochrome c was added (∼60%, *p* < 0.05), which was not observed when cells were co-treated with DPA (Figure 5B). This response occurred independently of changes in mitochondrial content, as there were no detectable differences in protein content of any of the mitochondrial OXPHOS complexes in myoblasts treated with PAL and/or DPA (Figures 6A–F).

## Discussion

This is the first study to compare the protective effects of EPA, DHA, as well as DPA against the lipotoxic effects of PAL in skeletal muscle cells. We demonstrate that PAL induces cell death as determined by changes in myoblast viability, nuclei number and nuclei morphology, in support of previous findings (Mandelkow et al., 2017; Saini et al., 2017). In addition, we determine that cell death occurs via apoptosis, as evidenced by increased TUNEL staining of PAL-treated myoblasts. Our results also indicate that EPA, DPA, and DHA protect against these deleterious effects of PAL, thereby allowing differentiation to occur, despite differences observed in ER stress signaling responses between each n-3 PUFA. Furthermore, we demonstrate that DPA mitigates PAL-induced reductions in mitochondrial membrane integrity, which is a potential mechanism underlying the observed protective effects of n-3 PUFAs. While the results of the present investigation align with previous data in differentiated myotubes showing that EPA and DHA elicit protection against the detrimental cellular effects of PAL (Bryner et al., 2012; Woodworth-Hobbs et al., 2014; Pinel et al., 2016), we show for the first time that DPA may elicit additional protective effects against PAL lipotoxicity (i.e., cell death and associated ER stress markers) relative to EPA and DHA.

The mechanisms underlying cell death in response to PAL are complex, multifaceted and involve multiple cell signaling pathways as demonstrated by Saini et al. (2017), who have shown that EPA protects against PAL-induced reductions in myoblast viability by reducing caspase activity. However, inhibiting caspase activity only partially prevents the cell death response, indicating other pathways such as the MAPK and JNK signaling may be involved. Additional mechanisms that may play a role in PAL-induced cell death include the induction of ER-stress signaling (Zhang E. et al., 2019), impaired autophagy processes involved in the removal of damaged mitochondria (Sadeghi et al., 2020), as well as sustained impairments in mitochondrial function (Rachek et al., 2007; Yuzefovych et al., 2010). Data in the present study align with the consensus that cellular PAL exposure results in lipotoxicity, and further demonstrates that n-3 PUFA treatments can ameliorate these deleterious effects thereby preventing cell death.

The present study also interrogated further mechanisms involving mitochondrial membrane integrity and cytochrome c release by which n-3 PUFAs may elicit protection against the lipotoxic effects of PAL leading to the observed reductions in myoblast viability, as investigated using complementary microscopy techniques to assess nuclear brightness and DNA fragmentation characteristic of apoptosis. Microscopy-based nuclei analysis confirmed that the PAL-induced reductions in nuclei per area occurred due to cell death and were not due to impairments in proliferation, as the number of cells initially plated was consistent between each experimental condition prior to analysis at ∼70% confluency. TUNEL stain binds to 3′-hydroxyl termini of DNA double strand breaks, a hallmark of the late stages of apoptosis. Unlike the microscopy-based analyses of nuclear area and perimeter, TUNEL analysis did not detect any changes in the percentage of apoptotic cells between individual n-3 PUFA and PAL co-treated cells. A limitation of the present study is that TUNEL staining was only analyzed at one time point. It is therefore difficult to ascertain precisely when apoptotic programmed cell death is occurring following PAL exposure. Nevertheless, the 16 h time point was selected as other investigations in both muscle and non-muscle cell types have demonstrated that PAL induces significant cell death at this time point (Listenberger et al., 2001; Hage Hassan et al., 2012; Salvado et al., 2013; Woodworth-Hobbs et al., 2017; Perry et al., 2018).

Results from studies undertaken in rodent and cell culture models both suggest that the provision of excess saturated fat can result in ER stress, leading to the activation of UPR signaling (Deldicque et al., 2010; Yuzefovych et al., 2013; Woodworth-Hobbs et al., 2017). The UPR involves the release of BiP from ER transmembrane signal transducers PKR-like ER kinase (PERK), inositol-requiring enzyme 1 (IRE1) and activating transcription factor 6 (ATF6). BiP is a key regulator of the UPR, since the activation of PERK, IRE1 and ATF6 is dependent on its dissociation from these proteins (Lee, 2005). Ultimately, these three arms of the UPR converge downstream on the transcription factor C/EBP homologous protein (CHOP), triggering apoptosis (Li et al., 2014). Given that PAL is known to induce ER stress and stimulate UPR signaling, it is unsurprising that BiP protein expression was elevated in response to PAL exposure. In an attempt to relieve ER stress, PERK activation results in an increase in eIF2α phosphorylation, suppressing protein translation and promoting proper protein folding and export from the ER (Harding et al., 2000). While we were unable to detect any changes in the phosphorylation status or protein content of eIF2α, given the transient nature of phosphorylation it is possible that eIF2α phosphorylation may have occurred at an earlier timepoint in an attempt to re-establish homeostasis after PAL treatment (Saelens et al., 2001). Considering the overall lack of consistency observed between the effects of each n-3 PUFA with respect to cell viability and ER stress markers, the beneficial protective effects of n-3 PUFAs on cell viability in the present study’s experimental design are unlikely to be due to protection against ER stress induction. The cell death and associated apoptosis responses observed do, however, align with that of the mitochondrial cytochrome c responses, indicating that PAL-induced cell death and the protective effects of n-3 PUFAs occur in a manner not solely dependent on the convergence of the UPR on CHOP, suggesting that alternative apoptotic pathways could lead to the observed reductions in viability (Green and Llambi, 2015). Nevertheless, PAL-induced cell death is attenuated when cells are co-treated with n-3 PUFAs. This response is most likely due to the inhibition of proteins responsible for the permeabilization of the outer mitochondrial membrane preventing the release of cytochrome c from mitochondria (Ott et al., 2007).

The results of the present study also complement other reports regarding the effects of n-3 PUFAs in promoting differentiation of viable skeletal muscle myoblasts to myotubes (Briolay et al., 2013; Saini et al., 2017). Despite differences in the timing of treatments and muscle cell line utilized, our results are in agreement with previous findings demonstrating that EPA and DHA promote differentiation of viable myoblasts to myotubes. Chronic treatment (up to 72 h) of differentiating L6 muscle cells with 20 μM EPA and DHA has also been shown to improve measures of differentiation (Briolay et al., 2013). In the current study we did not observe further increases in myogenin. In contrast, Briolay and co-workers found that both EPA and DHA increased the expression of myosin and creatine kinase as well as myogenic index to a greater extent compared to untreated control cells. Likewise, Saini et al. (2017) also provide evidence from mouse C2 cells that 50 μM EPA treatment for 72 h improves differentiation, as measured by creatine kinase and MyoD gene expression. In contrast, Zhang J. et al. (2019) and Hsueh et al. (2018) demonstrate that EPA and/or DHA can inhibit myoblast proliferation, reducing cell viability and myogenic potential resulting in attenuated expression of myogenin and myosin heavy chain mRNA expression as well as reduced myotube formation. There are a number of reasons why EPA and DHA in these two studies (Hsueh et al., 2018; Zhang J. et al., 2019) may show divergent effects to the results from the current investigation and previous work (Saini et al., 2017). Earlier studies have differentiated C2C12 cells in the presence of 50 μM EPA or DHA either in combination (100 μM PUFA total) (Hsueh et al., 2018), or separately for up to 72 h (Zhang J. et al., 2019). The 16 h single dose used in the current study allowed differentiation of viable myoblasts to occur, suggesting that chronic treatment with n-3 PUFAs in higher concentrations may have an inhibitory effect on differentiation. Nevertheless, the current study design is novel in that it directly compares the protective effects of the three n-3 PUFAs EPA, DPA, and DHA against the lipotoxic effects of PAL in myoblasts, as well as the successive effects on remaining viable myotubes within the same set of experiments.

Considering that DPA-PAL resulted in the greatest magnitude of protection against loss of cell viability when compared to PAL treated cells, changes in mitochondrial function and content were investigated as mechanisms that could potentially explain why cell viability was maintained despite remaining increases in ER stress signaling following DPA treatment. Several studies have proposed that the biological effects of n-3 PUFAs occur via improvements in mitochondrial bioenergetics (Aas et al., 2006; Vaughan et al., 2012; Lanza et al., 2013; Johnson et al., 2015). The protective effect of DPA against PAL-induced cell death may be explained by the incorporation of DPA into cellular membranes, increasing the level of unsaturated lipid species (e.g., phospholipids) and consequently increasing the fluidity of mitochondrial membranes thereby altering mitochondrial function (Sullivan et al., 2018). Increases in human mitochondrial membrane phospholipid species as a result of n-3 PUFA (EPA and DHA) supplementation have been shown to improve ADP kinetics rather than alter maximal mitochondrial respiration (Herbst et al., 2014). In support of these findings, we observed no increases in maximal mitochondrial respiration following DPA treatment. In contrast, we demonstrate that PAL compromises the integrity of outer mitochondrial membranes resulting in the release of cytochrome c. DPA attenuated this response, indicating that its incorporation into lipid membranes maintains the integrity of mitochondrial membranes despite the presence of PAL and associated increases in ER stress-related signaling. This is consistent with n-3 PUFA incorporation into mitochondrial membranes in human skeletal muscle observed following 12 week supplementation with EPA and DHA (Gerling et al., 2019). Given our data show increased cellular incorporation of DPA following incubation with 50 μM DPA, as well as elevated EPA levels that were not significantly different from DPA levels, further research focused on DPA’s mitochondrial incorporation and biological actions is warranted to determine how this dose and level of incorporation translates to *in vivo* models.

Future studies are warranted to dissect the precise direct versus indirect cellular mechanisms underlying n-3 PUFA mediated protection against PAL-induced lipotoxicity. Evidence suggests that n-3 PUFAs prevent the accumulation of PAL-derived ceramide and diglyceride lipid species known to elicit cytotoxic effects by increasing fatty acid oxidation (Capel et al., 2015; Pinel et al., 2016). One unexplored possibility of the current study is that the PAL treatment resulted in ceramide accumulation, causing excessive ROS production within the mitochondria. Consequently, this may have led to the activation of the mitochondrial permeability transition pore and release of cytochrome c responsible for the decrease in viability (Yuzefovych et al., 2012; Kim et al., 2017; Law et al., 2018). Additionally, n-3 PUFAs are known to be precursors to unique lipid mediators such as resolvins and protectins (Markworth et al., 2016; Serhan et al., 2018). Therefore, it is possible that the effects observed in the current study may have occurred indirectly via specific downstream cellular actions of their unique metabolites. Moreover, DPA may elicit distinct biological effects directly or via its conversion into lipid species that provide unique downstream actions and superior protection versus EPA and DHA against PAL-induced lipotoxicity observed in the current study. This indirect biological action of DPA is consistent with findings from Suphioglu et al. (2010) demonstrating that DHA protects against apoptosis in human neuronal cells indirectly via downstream actions on zinc transport that in turn reduce cellular zinc levels thereby promoting cell survival. It is possible that similar indirect n-3 PUFA cellular mechanism(s) could also be occurring in skeletal muscle in response to PAL-induced apoptosis. Considering the similar chemical structure of DPA relative to EPA and DHA, we speculate that the different biological effects of DPA are potentially explained by DPA being metabolized into different lipid species having unique properties and/or triggering distinct downstream cellular events than those produced by EPA and DHA. Our data support this hypothesis, as we did not observe an increase in DPA in response to EPA treatment, but did, however, observe retro-conversion of DPA to EPA following DPA treatment, demonstrating potential for DPA’s unique and superior biological effects.

In summary, the n-3 PUFAs EPA, DPA, and DHA elicit similar protective effects against PAL-induced lipotoxicity, thereby preventing apoptosis and promoting cell viability and differentiation of viable myoblasts to myotubes (Supplementary Figure S3). Furthermore, DPA maintains cell viability potentially via mitigating the loss of mitochondrial membrane integrity induced by PAL. Together, these data highlight the potential for n-3 PUFAs and specifically DPA to promote cell viability and combat deleterious effects of muscle cell lipid accumulation, as well as provides novel mechanistic underpinning for DPA’s protective effects against PAL-induced lipotoxicity.

## Data Availability Statement

All datasets presented in this study are included in the article/Supplementary Material.

## Author Contributions

BT, JW, JH, and NH contributed to the conception and design of the study, drafted and critically revised the manuscript, and approved the final submitted version of the manuscript. BT, JW, and NH performed and assisted with experiments, data analysis, and data interpretation.

## Conflict of Interest

The authors declare that the research was conducted in the absence of any commercial or financial relationships that could be construed as a potential conflict of interest.
