# Supplementary material for: Omega-3 Polyunsaturated Fatty Acids Mitigate Palmitate-Induced Impairments in Skeletal Muscle Cell Viability and Differentiation
Source: Front Physiol. 2020 Jun 3;11:563. doi: 10.3389/fphys.2020.00563 (PMC7283920; doi:10.3389/fphys.2020.00563)
Supplement: Supplementary file 1 [file Data_Sheet_1.docx]

Supplementary Material


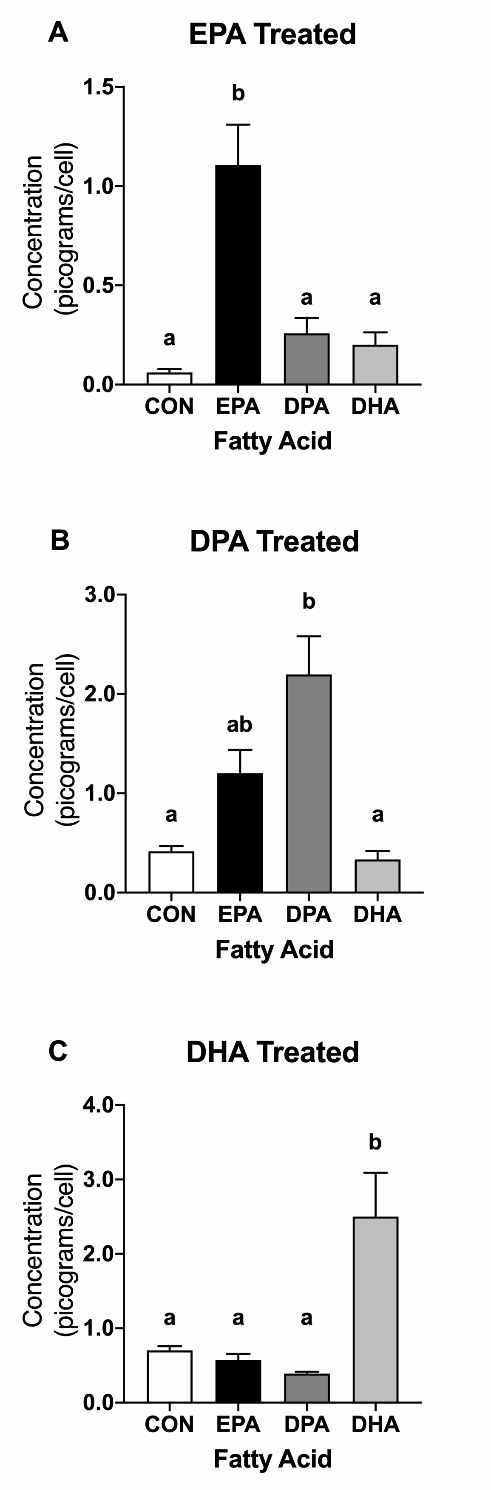


**Supplementary Figure 1**. **Treatment of C2C12 myoblasts with 50µM EPA, DPA or DHA increases the intracellular concentrations of each respective n-3 PUFA.** Cellular concentrations of EPA, DPA and DHA following 16h treatment with either ethanol vehicle control (CON), 50 µM EPA (A), 50 µM DPA (B) or 50 µM DHA (C). Fatty acid analysis was carried out by fatty acid methyl esters (FAME) analysis and data are presented as absolute concentrations relative to cell number. Treatments that do not share letters are considered statistically significant (p <0.05). Myoblasts were cultured to generate a total sample size of four technical replicates. Values are presented as mean ± SEM.

**
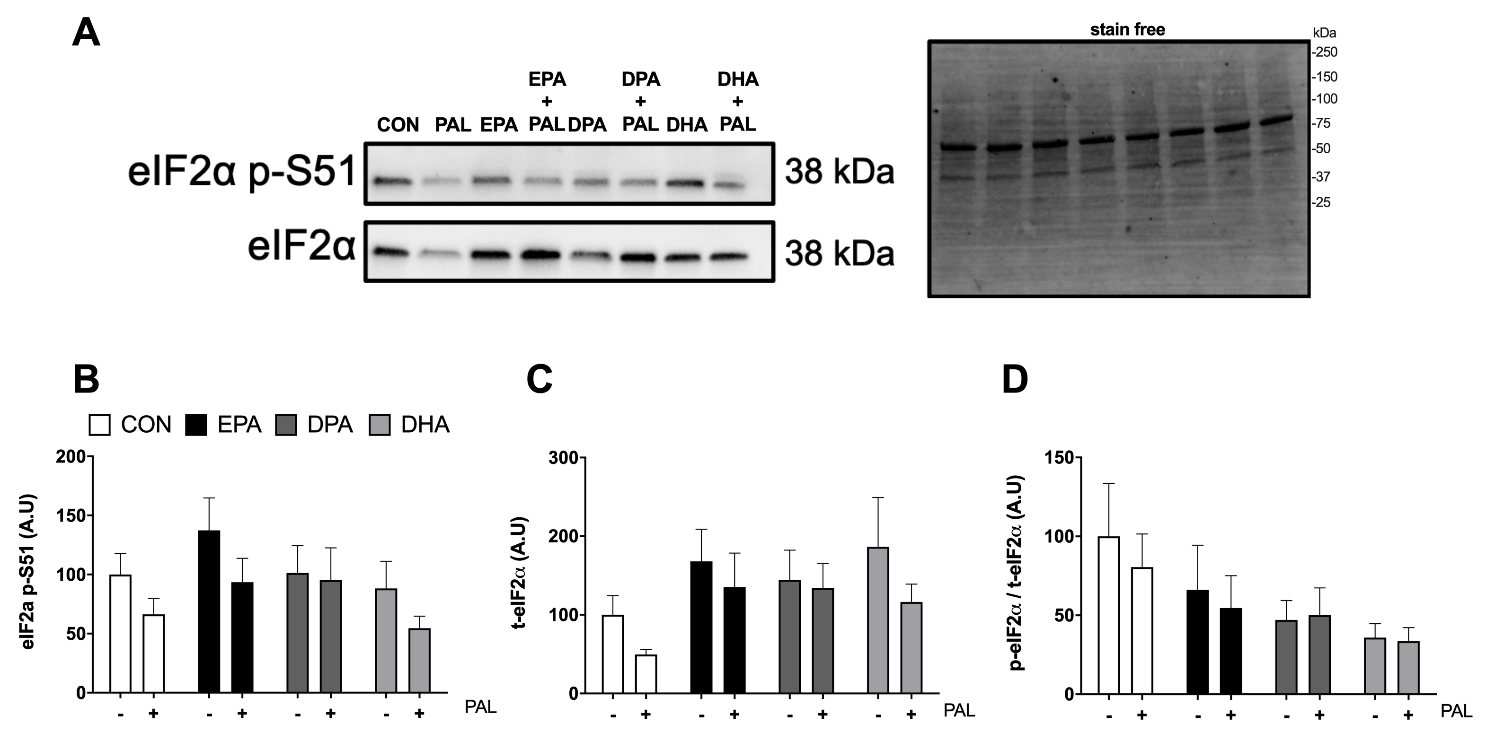
**

**Supplementary Figure 2**. **Total protein content and phosphorylation status of eIF2α is unchanged following PAL and/or n-3 PUFA treatment.** Representative images of total and phosphorylated eIF2α following 16h treatment with 500 μM PAL and/or 50 μM EPA, DPA or DHA, with a representative image of a stain free blot used for normalization (A). Quantified phosphorylation of eIF2α (Ser51) (B) and total protein content of eIF2α (C). Levels of phosphorylation expressed relative to total protein content of eIF2α (D). All densitometry values are expressed relative to protein content determined by stain free imaging and presented in arbitrary units relative to CON. Myoblasts were cultured and harvested independently to generate a total sample size of six biological replicates. Values are presented as mean ± SEM.

**
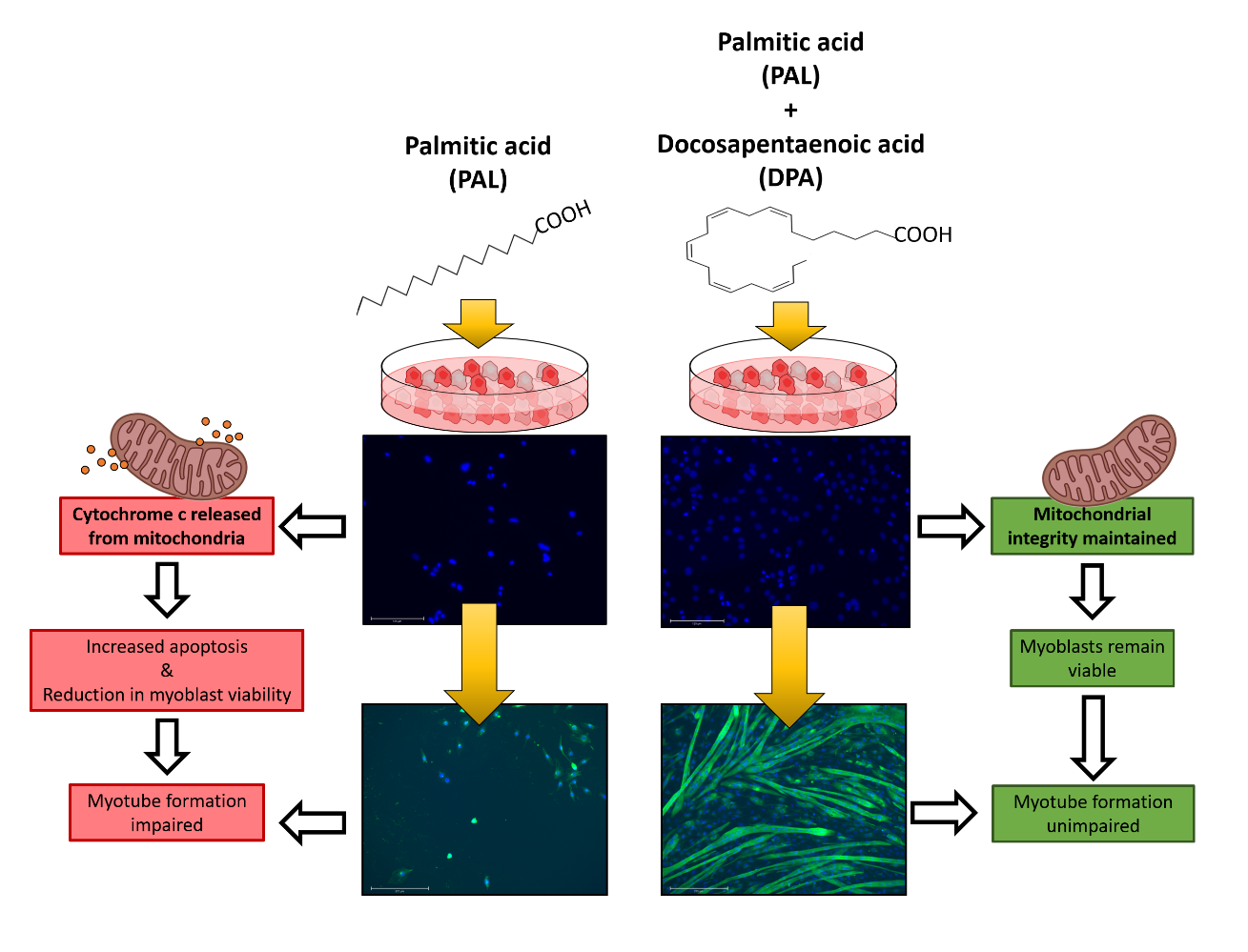
**

**Supplementary Figure 3**. Schematic diagram of the major novel findings and potential underlying mechanisms in this study. PAL reduces cell viability at 16 h and impairs myotube formation. All n-3 PUFAs (EPA, DPA and DHA) rescue myoblasts from the lipotoxic effects of PAL and allow myotube formation to occur. The maintenance of mitochondrial integrity is one possible mechanism, as depicted above for DPA, which may explain the protective effects of n-3 PUFAs against lipotoxic effects of PAL.
